# Real‐world assay variability between laboratories in monitoring of recombinant factor IX Fc fusion protein activity in plasma samples

**DOI:** 10.1111/ijlh.13189

**Published:** 2020-03-23

**Authors:** Jurg M. Sommer, Ali Sadeghi‐Khomami, Christopher Barnowski, Margareta Wikén, Annemieke J. Willemze

**Affiliations:** ^1^ Biogen Inc Cambridge MA USA; ^2^ Precision BioLogic Dartmouth NS Canada; ^3^ Sanofi Cambridge MA USA; ^4^ Swedish Orphan Biovitrum AB (Sobi) Stockholm Sweden

**Keywords:** chromogenic substrate assay, factor IX replacement therapy, haemophilia B, one‐stage clotting assay, recombinant factor IX Fc fusion protein

## Abstract

**Introduction:**

Monitoring of factor IX (FIX) replacement therapy in haemophilia B relies on accurate coagulation assays. However, considerable interlaboratory variability has been reported for one‐stage clotting (OSC) assays. This study aimed to evaluate the real‐world, interlaboratory variability of routine FIX activity assays used in clinical haemostasis laboratories for the measurement of recombinant FIX Fc fusion protein (rFIXFc) activity.

**Methods:**

Human FIX‐depleted plasma was spiked with rFIXFc at 0.80, 0.20 or 0.05 IU/mL based on label potency. Participating laboratories tested samples using their own routine OSC or chromogenic substrate (CS) assay protocols, reagents and FIX plasma standards. Laboratories could perform more than one measurement and method, and were not fully blinded to nominal activity values.

**Results:**

A total of 142 laboratories contributed OSC results from 175 sample kits using 11 different activated partial thromboplastin time (aPTT) reagents. The median recovered FIX activity for the 0.80, 0.20 and 0.05 IU/mL samples was 0.72 IU/mL, 0.21 IU/mL and 0.060 IU/mL, respectively. Across all OSC reagents, interlaboratory variability (% CV) per aPTT reagent ranged from 9.4% to 32.1%, 8.2% to 32.6% and 12.2% to 42.0% at the 0.80, 0.20 and 0.05 IU/mL levels, respectively. CS results showed excellent median recoveries at all nominal levels (87.5% to 115.0%; n = 11) with low interlaboratory variability (CV 3.6% to 15.4%).

**Conclusion:**

This large, real‐world data set indicates that rFIXFc activity in plasma samples can be accurately measured with the majority of routine OSC and CS assay methods. Given the variation in FIX assay procedures between sites, it is important that individual laboratories qualify their in‐house methods for monitoring of rFIXFc activity.

## 1 INTRODUCTION

Infusion of replacement factor IX (FIX) is the preferred treatment for haemophilia B. The pharmacokinetics of FIX replacement appear more complex than factor VIII (FVIII) replacement in haemophilia A.^1^, ^2^, ^3^ Preclinical studies in animal models suggest the presence of a significant noncirculating extravascular pool of FIX that can be mobilized when needed.^4^, ^5^, ^6^, ^7^ In fact, FIX has been shown to cross the endothelium and bind tightly and reversibly to type IV collagen in the extracellular matrix.^4^, ^8^, ^9^ However, although FIX levels in plasma may not be the only factor determining bleed protection in haemophilia B, current clinical practice relies on the accurate measurement of FIX activity in plasma in order to monitor treatment and deliver optimal care to patients.

The burden of frequent infusions required for effective prophylaxis has been reduced considerably by the introduction of extended half‐life (EHL) recombinant FIX (rFIX) products.^10^, ^11^, ^12^ However, structural and chemical modifications in EHL rFIX products may alter their assay behaviour compared to conventional rFIX products.^13^ For instance, there is some evidence that the addition of long‐chain polyethylene glycol (PEG) in certain EHL products may lead to significant over‐ or underestimation of one‐stage clotting (OSC) assay results.^14^, ^15^ Haemophilia treaters and specialty coagulation laboratories may justifiably have concerns as to whether their routine FIX assay can be used to accurately monitor an EHL rFIX product in patients.

The OSC assay is used for potency assignment of all current FIX products and is also the most commonly used method for determining circulating levels of FIX activity in human plasma samples.^16^, ^17^ The assay is based on the activated partial thromboplastin time (aPTT), which compares the clot time of a patient sample against that of a calibrator FIX product when both are diluted into a FIX‐depleted substrate plasma.^18^ Calibrators are typically composed of pooled normal human plasma lots with an activity assigned against a FIX reference product, such as the World Health Organization (WHO) International Standard for FIX plasma.^19^ Historically, clinical FIX assay discrepancies have not been a major concern in the testing of conventional rFIX products. However, more recently, proficiency studies and manufacturer field studies have shown different levels of variability in measuring both rFIX and EHL rFIX products against the plasma FIX reference.^15^, ^20^, ^21^, ^22^, ^23^

One of the major reasons for interlaboratory variability in FIX measurements is the use of a large variety of aPTT reagents,^22^ which can contain different types of contact activators (various forms of micronized silica, kaolin, ellagic acid or polyphenols) and different composition and sources of phospholipids (synthetic, plant‐ or animal‐derived). Additional interlaboratory variability may be introduced when these reagents are mixed with FIX‐depleted substrate plasma from different sources, subjected to further differences in assay protocols and analysed on different instruments.^24^

The accuracy of OSC reagents for monitoring recombinant FIX Fc fusion protein (rFIXFc; Alprolix^®^), compared to a conventional rFIX product, was evaluated in a previous field study of 30 specialty coagulation laboratories. The study found that the OSC assay could be used to measure rFIXFc with acceptable accuracy and reliability, although measurements appeared to some extent to be aPTT reagent‐dependent, as lower values were obtained when using silica‐ or kaolin‐based activators compared to ellagic acid‐based activators.^21^ However, the number of laboratories using the same instruments and reagents was limited, preventing differentiation of the effect of the individual reagents on assay performance from general assay variability.

The objective of this real‐world study was to evaluate the accuracy of routine FIX activity assays used by individual clinical haemostasis laboratories for the measurement of rFIXFc activity in spiked plasma samples. This was one of the largest field studies to date for any coagulation factor replacement therapy on the ability of commonly used OSC and CS assays to measure factor activity, which allowed for a comprehensive assessment of the real‐world performance of individual reagents and analysers and their impact on assay variability.

## 2 MATERIALS AND METHODS

### 2.1 Laboratory field study kit preparation

Two separate lots of rFIXFc‐spiked sample kits were prepared by Precision BioLogic using identical methods and distributed (Precision BioLogic) to participating clinical haemostasis laboratories. One lot was provided (by Biogen) to laboratories in Australia, Canada, New Zealand and the USA from January 2014 to May 2015, while another lot was provided (by Sobi) to laboratories in Croatia, Finland, France, Germany, Ireland, Italy, Netherlands, Slovenia, Sweden, Switzerland and the UK from August 2016 to December 2018. FIX‐immunodepleted plasma with no measurable FIX activity (<0.01 IU/mL) was prepared by Precision BioLogic and spiked with rFIXFc drug substance to a nominal FIX activity of 0.80 IU/mL (Sample A), 0.20 IU/mL (Sample B) and 0.05 IU/mL (Sample C). The potency of the rFIXFc drug substance was assigned by Biogen using a Sysmex CA‐1500 analyser (Siemens) and the Siemens Actin aPTT reagent against in‐house rFIXFc reference material that had its potency originally assigned against the 3rd International Standard for FIX concentrate (96/854).^25^

Samples were distributed as frozen plasma aliquots, rather than in lyophilized format, which more closely resembles patient plasma samples stored in laboratories for routine testing and mitigates pre‐analytical variability associated with sample reconstitution. A comparison of FIX activity between the two lots was performed to verify that data from both lots could be pooled for analysis. In‐house evaluation and field study results did not indicate differences that would significantly impact the merging of data obtained from the Sobi and Biogen kits (analysis not shown). Stability studies were conducted by Precision BioLogic on sample kits stored at ultra‐low temperatures (<−70°C) during the course of each field study to ensure rFIXFc activity was preserved.

### 2.2 Study design

Participating laboratories received kits containing 1‐mL frozen vials of plasma samples spiked at each of the three nominal rFIXFc activity levels for testing using their own routine FIX assay procedures, analysers and calibrators. Laboratories could perform more than one measurement or method of FIX activity assay and were provided with additional sample kits when requested. As most laboratories are already regulated by external quality assurance programmes, the purpose of this study was to allow individual laboratories to gauge the accuracy of their own FIX activity assay(s) and controls for monitoring rFIXFc activity in plasma samples. Assay controls and FIX comparator products were therefore not provided by the study sponsors, and laboratories were not fully blinded to nominal activity values (vials were not labelled with nominal activity; this information was provided to the site contact/laboratory director, but may not have been known to the person performing the assays).

Data requested from participating laboratories included model and manufacturer of the coagulation analyser, software version, aPTT reagent and manufacturer, calibrator plasma and manufacturer, diluent and manufacturer, FIX‐depleted or deficient plasma manufacturer, protocol name or number, calibration frequency, number of dilutions tested per sample, clot times, type of regression analysis and calculated FIX activity.

### 2.3 Data analysis

Two data sets were excluded from the analysis: one due to testing errors by the participating laboratory and one from a laboratory using a semi‐automated analyser (Diagnostica Stago STart). Spike recovery is defined as the recovered percentage of nominal activity (ie 0.80, 0.20, 0.05 IU/mL). Descriptive statistics and the Mann‐Whitney test (GraphPad Prism) were used to summarize the performance of individual reagents, analysers or impact of specific method parameters.

## 3 RESULTS

### 3.1 Participating laboratories

In total, 142 laboratories from Europe, USA, Canada, Australia and New Zealand participated in the study. Sixty‐eight laboratories using the Biogen lot provided 74 data sets measured with the OSC assay. Seventy‐four participants using the Sobi lot provided a total of 101 OSC data sets and 11 CS data sets. Across all laboratories, a total of 11 different aPTT reagents were used (Table S1); SynthASil was the most commonly used (n = 37 samples), followed by CK Prest (n = 33) and Actin FS (n = 30).

### 3.2 One‐stage clotting assay: overall results

**FIGURE 1:**
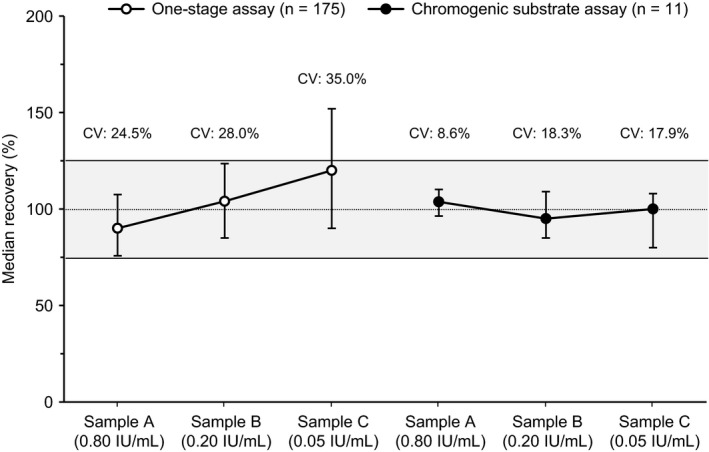
Overall recovery of nominal potency in rFIXFc samples using the one‐stage clotting assay (n = 175 data sets) and the chromogenic substrate assay (n = 11 data sets). Error bars show interquartile range. Shaded area shows the ± 25% recovery range of nominal activity. CV, coefficient of variation; IU, international units; rFIXFc, recombinant factor IX Fc fusion protein

Across the different methods used by the 142 participating laboratories, the median (range) FIX activity observed in 175 independent tests of the 0.80 IU/mL sample was 0.72 (0.21‐1.37) IU/mL. FIX activity observed for the 0.20 and 0.05 IU/mL samples was 0.21 (0.08‐0.42) IU/mL and 0.060 (0.020‐0.137) IU/mL, respectively (Table 1). For all data sets, the overall coefficient of variation (CV) for the 0.80, 0.20 and 0.05 IU/mL samples was 24.5%, 28.0% and 35.0%, respectively (Figure 1).

**TABLE 1:** One‐stage clotting assay results by aPTT reagent for plasma samples spiked with rFIXFc at 0.80, 0.20 and 0.05 IU/mL

	aPTT reagent	Actin FS	Actin FSL	Cephascreen	Pathromtin SL	SynthASil	APTT‐SP	Triniclot APTT‐S	Triniclot APTT‐HS	PTT‐A	PTT‐LA	CK Prest	All reagents
Sample A (0.80 IU/mL)	Total (n)	30	12	6	11	37	8	7	8	22	1	33	175
	Sobi (n)	20	1	4	11	21	6	0	3	8	1	26	101
	Biogen (n)	10	11	2	0	16	2	7	5	14	0	7	74
	Median (IU/mL)	0.945	0.886	0.774	0.730	0.805	0.631	0.710	0.683	0.611	0.714	0.570	0.720
	Median recovery	118.1%	110.8%	96.8%	91.3%	100.6%	78.8%	88.8%	85.3%	76.4%	89.3%	71.3%	90.0%
	Range (IU/mL)	0.606‐1.365	0.548‐1.120	0.670‐0.973	0.640‐0.940	0.420‐0.926	0.206‐0.870	0.620‐0.820	0.599‐0.935	0.380‐0.720	na	0.360‐0.705	0.206‐1.365
	Mean (IU/mL)	0.944	0.883	0.800	0.785	0.777	0.604	0.724	0.704	0.590	0.714	0.549	0.734
	CV	15.1%	16.9%	12.9%	14.0%	13.5%	32.1%	9.4%	15.9%	15.2%	na	16.2%	24.5%
Sample B (0.20 IU/mL)	Total (n)	30	12	6	11	37	8	7	8	22	1	33	175
	Sobi (n)	20	1	4	11	21	6	0	3	8	1	26	101
	Biogen (n)	10	11	2	0	16	2	7	5	14	0	7	74
	Median (IU/mL)	0.244	0.275	0.223	0.250	0.220	0.165	0.220	0.205	0.170	0.238	0.150	0.208
	Median recovery	122.0%	137.5%	111.5%	125.0%	110.0%	82.5%	110.0%	102.5%	85.0%	119.0%	75.0%	104.0%
	Range (IU/mL)	0.148‐0.420	0.155‐0.350	0.220‐0.268	0.177‐0.363	0.110‐0.300	0.080‐0.260	0.190‐0.250	0.177‐0.294	0.097‐0.220	na	0.100‐0.188	0.080‐0.420
	Mean (IU/mL)	0.253	0.269	0.232	0.262	0.216	0.162	0.217	0.214	0.167	0.238	0.152	0.209
	CV	18.8%	22.4%	8.2%	26.8%	19.3%	32.6%	10.2%	18.0%	20.6%	na	15.6%	28.0%
Sample C (0.05 IU/mL)	Total (n)	30	12	6	11	37	8	7	8	22	1	33	175
	Sobi (n)	20	1	4	11	21	6	0	3	8	1	26	101
	Biogen (n)	10	11	2	0	16	2	7	5	14	0	7	74
	Median (IU/mL)	0.070	0.079	0.062	0.065	0.066	0.049	0.070	0.070	0.042	0.074	0.045	0.060
	Median recovery	140.0%	158.0%	123.0%	130.0%	132.0%	97.0%	140.0%	140.0%	83.0%	148.0%	90.0%	120.0%
	Range (IU/mL)	0.041‐0.137	0.030‐0.120	0.060‐0.080	0.030‐0.120	0.020‐0.100	0.030‐0.090	0.050‐0.100	0.055‐0.087	0.020‐0.090	na	0.030‐0.070	0.020‐0.137
	Mean (IU/mL)	0.078	0.079	0.065	0.070	0.063	0.052	0.071	0.072	0.047	0.074	0.047	0.062
	CV	25.6%	34.1%	12.2%	42.0%	28.9%	35.6%	24.4%	18.3%	38.7%	na	21.0%	35.0%

Abbreviations: aPTT, activated partial thromboplastin time; CV, coefficient of variation; IU, international units; na, not applicable; rFIXFc, recombinant factor IX Fc fusion protein.

### 3.3 One‐stage clotting assay: comparison of aPTT reagents

**FIGURE 2:**
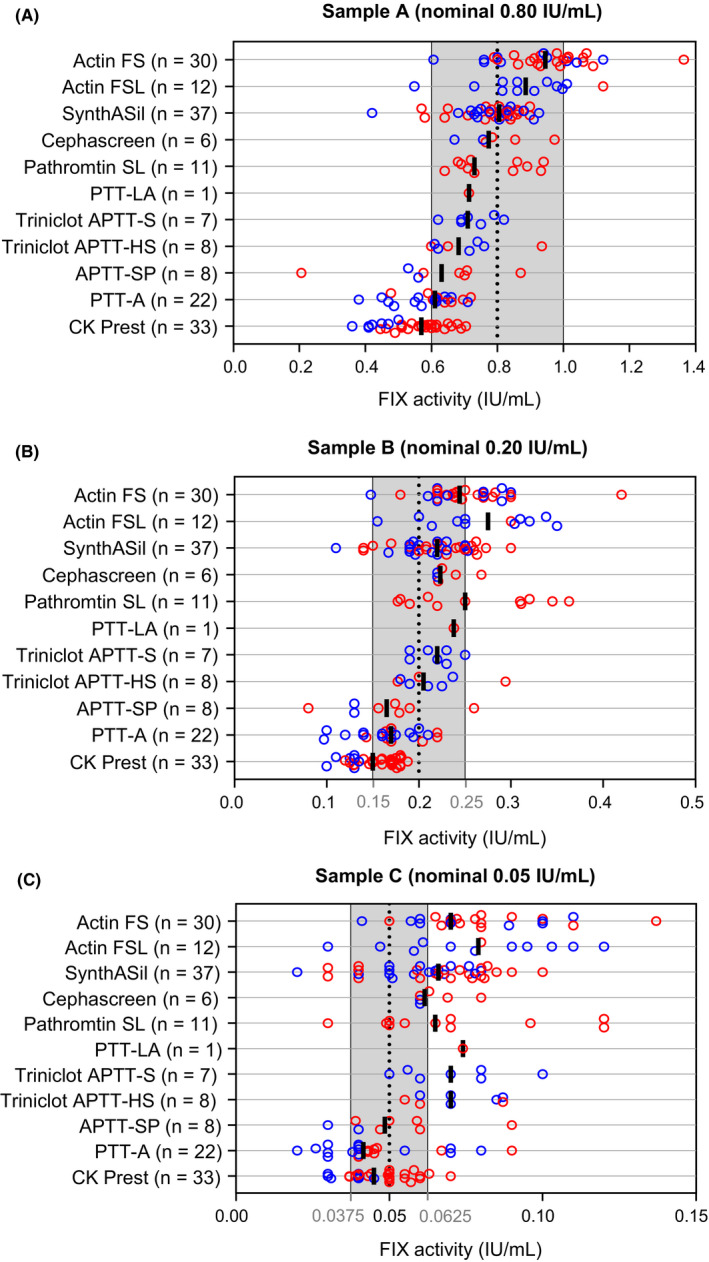
One‐stage clotting assay results for plasma samples spiked with rFIXFc at nominal levels of (A) 0.80 IU/mL, (B) 0.20 IU/mL or (C) 0.05 IU/mL. Data points are colour‐coded according to whether they were derived from a Sobi (red) or Biogen spiked sample lot (blue). Black bars shown the median FIX activity (IU/mL) for each aPTT reagent. Shaded areas show the ± 25% recovery range of the nominal activity (dotted vertical line). aPTT, activated partial thromboplastin time; FIX, factor IX; IU, international units; rFIXFc, recombinant factor IX Fc fusion protein

OSC results for all reagents are shown in Figure 2. The interlaboratory CV for individual reagents was generally lower than the overall variability, ranging from 9.4% to 32.1% for the 0.80 IU/mL sample, 8.2% to 32.6% for the 0.20 IU/mL sample and 12.2% to 42.0% for the 0.05 IU/mL sample (Table 1). For the 0.80 IU/mL sample, the median FIX activity observed for all reagents was within 75‐125% of the nominal activity, with the exception of the Stago CK Prest reagent (kaolin activator), which showed 71.3% of the expected FIX activity (Table 1). For the 0.20 IU/mL sample, median recovery was within 75‐125% of nominal activity for all but one reagent (Actin FSL). For the 0.05 IU/mL sample, four reagents had median recoveries within 75‐125% of the nominal values (Cephascreen, APPT‐SP, PTT‐A and CK Prest), while the remaining seven demonstrated between 30.0% and 58.0% over‐recovery (Table 1, Figure 2).

The reagents showing a median recovery closest to nominal activity varied between the different levels: these included Cephascreen, Pathromtin SL and SynthASil at the 0.80 IU/mL level; Cephascreen, SynthASil and the two Triniclot reagents (APTT‐S and APTT‐HS) at the 0.20 IU/mL level; and Cephascreen, APTT‐SP, PTT‐A and CK Prest at the 0.05 IU/mL level (Table 1, Figure 2). The ellagic acid‐based aPTT reagents from Siemens (Actin FS and Actin FSL) demonstrated median over‐recovery at all levels (Table 1). Conversely, the 33 data sets using the kaolin‐based reagent CK Prest showed median under‐recovery at all levels, with CVs ranging from 16% to 21% (Table 1). Notably, when using CK Prest, the median recovery results from 7 Biogen data sets from US and Canadian laboratories were significantly lower than the 26 results obtained from European laboratories (Sobi lot) for all three nominal concentrations (52.5% vs 73.3%, Mann‐Whitney *P* < .0001; 65.0% vs 82.5%, *P* = .0003; and 80.0% vs 100.0%, *P* = .0026 for the 0.80, 0.20 and 0.05 IU/mL levels, respectively); these differences can be visualized in Figure 2.

### 3.4 One‐stage clotting assay: other variables

In addition to the aPTT reagent, other possible contributors to OSC assay interlaboratory variability include variations in procedure, instruments, the diluent used, dilution method and source of the assay calibrator.

While most of the laboratories included in this study used a lyophilized FIX plasma calibrator provided by the instrument manufacturer, 12 laboratories (8.5%) used a frozen plasma calibrator from a different provider, or in one case, an in‐house preparation of normal pooled plasma. Several laboratories also reported the use of alternate (ie not recommended by the analyser manufacturer) aPTT reagents (11.3%), calibrators (13.2%), FIX‐deficient plasma substrates (25.8%) and diluents (2.5%).

While there were generally only minor differences when using a particular aPTT reagent on various instruments from the same manufacturer (Table S2), using a reagent on an instrument from another manufacturer in some cases resulted in significant differences. For example, an assay using SynthASil performed on a Sysmex instrument resulted in approximately 20% lower median activity at the 0.80 IU/mL level compared to using this reagent in a manufacturer‐approved method on an Instrument Laboratory analyser (*P* = .004).

Calibration frequency was also evaluated as a potential covariate in determining the accuracy of OSC assay results. A wide range of calibration frequencies was reported by participating laboratories (Table S3), and a trend towards lower percentage error was observed with more frequent calibration among all results combined. However, an improvement in accuracy with increased calibration was not observed when evaluating individual analysers or APTT reagents.

With regard to multidilution analysis (MDA), approximately half of all laboratories tested only a single dilution of each sample rather than performing a recommended MDA (Table S4). Overall, no trend towards either improvement or worsening in accuracy was observed by laboratories performing MDA.

### 3.5 Chromogenic substrate assay

Nine laboratories testing the Sobi lot also performed FIX CS assays using one or both of the two commercially available kits, providing 11 CS data sets in total: 8 using the Hyphen Biophen (Hyphen BioMed) and 3 using the Rossix FIX kit (Rossix AB). Good recovery and relatively low interlaboratory variability were observed for both CS kits when adapted to an Instrument Laboratory TOP, Siemens BCS, Sysmex, or Diagnostica Stago instrument calibrated against the manufacturer's or a third‐party FIX plasma calibrator, with potency typically assigned by the OSC. Median recovery was 100.0% and 104.5% of expected activity at the 0.80 IU/mL level for the Hyphen Biophen and Rossix CS kits, respectively (Table 2). Both CS kits showed excellent dilution linearity and did not overestimate FIX activity at lower nominal rFIXFc concentrations (Figure 1), with median spike recoveries of 87.5% and 115.0% at the 0.20 IU/mL level, and 90.0% and 108.0% at the 0.05 IU/mL level (Table 2).

**TABLE 2:** Chromogenic assay results from 11 data sets

Reagent manufacturer	Sample A (nominal: 0.80 IU/mL)		Sample B (nominal: 0.20 IU/mL)		Sample C (nominal: 0.05 IU/mL)	
	Hyphen BioMed	Rossix AB	Hyphen BioMed	Rossix AB	Hyphen BioMed	Rossix AB
N	8	3	8	3	8	3
Median FIX activity (IU/mL)	0.800	0.836	0.175	0.230	0.045	0.054
Median recovery	100.0%	104.5%	87.5%	115.0%	90.0%	108.0%
Range (IU/mL)	0.68‐0.89	0.83‐0.89	0.15‐0.22	0.22‐0.27	0.039‐0.058	0.050‐0.067
Mean FIX activity IU/mL)	0.803	0.851	0.178	0.239	0.046	0.057
CV	9.7%	3.6%	12.1%	11.7%	15.4%	15.2%

Note

Hyphen analysers: Siemens, Sysmex, Stago, Instrument Laboratory; Rossix analysers: Siemens, Instrument Laboratory.

Abbreviations: CV, coefficient of variation; FIX, factor IX; IU, international units.

## 4 DISCUSSION

The most effective way to evaluate the real‐world assay performance of a new rFIX product is through field studies that include a sufficient number of laboratories to obtain consensus on assay results for commonly used aPTT reagents and coagulation analysers. Rather than investigating general proficiency in performing FIX assays, the aim of this study was to allow laboratories to evaluate the accuracy of their routine FIX assays and controls for determining rFIXFc activity. A FIX comparator product was therefore not provided to participating laboratories. This real‐world study evaluated rFIXFc monitoring based on 175 OSC data sets provided by 142 clinical haemostasis laboratories located across Europe, USA, Canada, Australia and New Zealand (providing information on 11 widely used aPTT reagents), as well as 11 CS assay data sets provided by nine laboratories (providing information on two FIX chromogenic kits).

Across all laboratories, the overall OSC variability at the 0.80 IU/mL level was within a generally acceptable 75‐125% recovery level (CV 24.5%) and was within the range of variability observed for conventional rFIX products when assayed against a plasma‐derived FIX standard.^26^ Progressively higher recovery was observed for most aPTT reagents at lower FIX levels. This tendency towards over‐recovery at lower FIX levels is not a unique characteristic of rFIXFc, since similar findings have been reported in previous field studies of other FIX products, including conventional rFIX,^21^ rFIX albumin fusion protein^27^ (rIX‐FP; Idelvion^®^) and pegylated rFIX^28^ (N9‐GP; Refixia^®^). It is important to note, however, that thus far there has been no consensus on what constitutes an ‘acceptable’ level of accuracy and different studies have specified different thresholds. For instance, previous field studies evaluating N9‐GP or rIX‐FP activity using the OSC assay have used a ±30% acceptance criteria for accuracy (corresponding to 70‐130% recovery).^23^, ^27^, ^28^ Given the greater variability observed at lower FIX levels, it may be necessary to reconsider what constitutes an acceptable threshold at such levels.

An approximately two‐fold overestimation at low levels of FIX (0.03 IU/mL) was also observed in a recent field study that measured FIX activity in samples spiked with a plasma‐derived FIX product, demonstrating that the overestimation is not due to an intrinsic property of recombinant FIX products.^29^ The approximately 30% higher rFIXFc recovery at 0.05 IU/mL vs 0.80 IU/mL by most aPTT reagents is therefore likely caused by the assay calibration method. The use of buffer as a diluent for the FIX calibrator has been known to produce discordant results at low levels^30^; this has also been shown recently in FVIII studies.^31^ Previous research has suggested that dilution of the FIX assay calibrator in buffer by the coagulation analyser could be the main reason for the progressive overestimation of lower nominal FIX levels, as this effect can be largely avoided by having the calibrator diluted in FIX‐depleted plasma (Buyue and Sommer; unpublished data).

This study identified the aPTT reagent as a major contributor to interlaboratory variation in rFIXFc OSC assay results. We also identified a few cases where using a reagent on an instrument from a different manufacturer resulted in significant differences compared to the manufacturer‐approved method. However, due to methodological differences between manufacturers (eg differences in assay protocol, diluent or calibrators), the impact of these variables on OSC results could not be separated from the reagent‐specific impact on the assay. While 34.6% of assays were performed using at least one component which was not recommended by the manufacturer of the analyser, numbers were too low to permit further analysis. Evaluation of instrument calibration frequency and MDA on accuracy of overall results did not identify a consistent impact on OSC results; however, as the study was not designed to evaluate these, it is highly likely that these are confounding factors in the analysis.

In a previous rFIXFc field study (30 laboratories), a generally lower recovery of rFIXFc by silica‐ and kaolin‐based reagents was reported compared to ellagic acid‐based activators.^21^ However, the previous study may have oversimplified the assessment of assay performance by grouping results by reagent type. In the present study, ellagic acid‐containing reagents (Actin FS, Actin FSL) showed over‐recovery at all levels, as shown previously,^21^ while reagents containing polyphenols (Cephascreen) and some containing silica‐based activators (SynthASil, Pathromtin SL) provided results within 5% of nominal activity at the 0.80 IU/mL level (Table S2).

The aPTT reagent CK Prest, which uses kaolin as activator, was previously reported to underestimate rFIXFc activity, with approximately 50% recovery at the 0.80 IU/mL spike level, based on data sets from four laboratories.^21^ Underestimation in CK Prest OSC assay performance has also been observed for rIX‐FP^32^ and N9‐GP.^14^ Although CK Prest showed a trend for under‐recovery in the present study, particularly at higher spike levels, the degree of accuracy reported across all laboratories that used this reagent was substantially higher compared to the previous study. Interestingly, when looking specifically at the CK Prest results reported by the seven laboratories located in the United States and Canada, the 53% spike recovery observed for the 0.80 IU/mL sample was in line with previously published results,^21^ while a 73% spike recovery for the 0.80 IU/mL samples was observed in the 26 samples from European laboratories. This discrepancy in CK Prest assay performance between North America and Europe may be due to slightly different protocols or other assay reagents, such as different FIX‐deficient plasmas marketed by Diagnostica Stago in North America vs Europe.

Data from the nine laboratories that contributed 11 CS assay data sets showed a relatively high level of accuracy in the measurement of rFIXFc with the two commercially available CS kits for FIX, with median spike recoveries within 15% of the nominal activity, and interlaboratory CV within 19%. Contrary to a previous report,^33^ minimal discordance was observed in CS results between the Hyphen and Rossix kits at the 0.80 IU/mL level. However, at lower FIX levels, recovery tended to be higher using the Rossix kit, in line with previous studies.^32^, ^33^, ^34^, ^35^ Although only a limited number of samples were tested, these results suggest that the CS assay is a promising option for monitoring of rFIXFc activity in plasma samples. Previous studies have reported good correlation between assigned potency and CS activity for nonacog gamma (rFIX; Rixubis^®^)^20^ and the PEGylated rFIX product N9‐GP in plasma.^28^ By contrast, studies have shown a tendency for the CS assay to underestimate the activity of nonacog alfa (rFIX; BeneFIX^®^) by approximately 20% compared to the OSC assay^20^, ^36^ and to exhibit large discrepancies relative to OSC potency assignment for rIX‐FP.^37^ Nonetheless, this study reported close agreement between OSC potency assessment of rFIXFc (using the Siemens Actin reagent) and its CS FIX activity; as more laboratories add the CS assay to their line‐up of FIX tests, this result is reassuring.

The samples used in this study consisted of FIX‐immunodepleted plasma spiked in vitro with rFIXFc. Previous field studies of EHL rFIX products have similarly used in vitro spiked samples,^21^, ^23^, ^27^ despite concerns that spiked samples may behave differently to post‐infusion (ex vivo) plasma samples from patients with haemophilia B, particularly as product from ex vivo samples would have been circulating in the body for several hours or days and potentially subjected to molecular modifications. Nonetheless, it has been shown that plasma samples spiked with FIX products^37^, ^38^ (Replenine^®^, rFIX) behave similarly to postinfusion patient samples in OSC and CS assays. Currently, no results have been published on the commutability of rFIXFc spiked plasma with postinfusion rFIXFc plasma samples in FIX activity assays. However, given the size of this study, it would have been very challenging to distribute patient plasma samples collected at similar timepoints postinfusion to all participating laboratories; this would have also introduced an extra source of individual variability to the data sets. Therefore, the use of rFIXFc‐spiked samples is considered a reasonable approach for this large, real‐world study.

## 5 CONCLUSION

This extensive evaluation of the real‐world performance of OSC FIX assays used in clinical haemostasis laboratories demonstrated that, despite interlaboratory variability among aPTT reagents, rFIXFc levels can be reliably assessed by the majority of OSC in‐house assay procedures. Overall, while average results for the 11 different aPTT reagents were close to nominal activity, a considerable number of laboratories contributed results outside the 75‐125% range of nominal recovery. Significant variability between results of laboratories reporting the same method also highlights the need for laboratories to better qualify their in‐house OSC assay procedures and possibly apply more rigorous assay controls. Of note, the 73% mean spike recovery for the 26 0.80 IU/mL samples analysed in Europe using the CK Prest reagent was much improved compared to the previous report^21^ (53%) and the stated recovery in the rFIXFc package insert, both of which were based on measurements from US and Canadian laboratories.

Although fewer measurements using the CS assay were contributed, the data from this study suggest that use of either of the two commercially available CS FIX assay kits is promising for the monitoring of plasma rFIXFc activity, particularly for measurement of low FIX activities. Although generally higher recoveries of rFIXFc activity using the Rossix kit compared to the Hyphen Biomed kit have been observed in several studies, a larger number of CS data sets will still be needed to determine whether either of these kits is in fact superior for the accurate measurement of rFIXFc activity. CS assay users should therefore continue to verify their own assay methodology for use with rFIXFc.

## CONFLICTS OF INTEREST

JS: Employee of Biogen at the time of the study; consultancy fees from Sobi; ASK: Employee of Precision BioLogic; CB: Employee of Sanofi Genzyme; MW: Employee of Sobi at the time of the study; AW: Employee of Sobi at the time of the study; current employee of Sanofi.

## AUTHOR CONTRIBUTIONS

JS, ASK and MW contributed substantially to study conception and design; JS, ASK, CB, MW and AW contributed substantially to analysis and interpretation of the data; JS, ASK, CB, MW and AW drafted the article or revised it critically for important intellectual content; and JS, ASK, CB, MW and AW approved the final version of the article to be published.

## Supporting information

Table S1‐S4

## Data Availability

Sobi is committed to responsible and ethical sharing of data on participant level and summary data for medicines and indications approved by EMA and/or FDA, while protecting individual participant integrity and compliance with applicable legislation. Data access will be granted in response to qualified research requests. All requests are evaluated by a cross‐functional panel of experts within Sobi and a decision on sharing will be based on the scientific merit and feasibility of the research proposal, maintenance of personal integrity and commitment to publication of the results. To request access to study data, a data sharing request form (available on www.sobi.com) should be sent to medical.info@sobi.com.
