# Supplementary material for: Real‐world assay variability between laboratories in monitoring of recombinant factor IX Fc fusion protein activity in plasma samples
Source: Int J Lab Hematol. 2020 Mar 23;42(3):350–8. doi: 10.1111/ijlh.13189 (PMC7318191; doi:10.1111/ijlh.13189)
Supplement: Supplementary file 1 — Table S1‐S4 [file IJLH-42-350-s001.docx]

SUPPLEMENTARY APPENDIX

**Table S1.** Activated partial thromboplastin time (aPTT) reagents used by participating clinical haemostasis laboratories

| **aPTT Reagent** | **Manufacturer** | **Activator** | **Phospholipid source** |
| --- | --- | --- | --- |
| Actin FS | Siemens | Ellagic acid | Purified soy |
| Actin FSL | Siemens | Ellagic acid | Purified soy and rabbit brain |
| APTT-SP | HemosIL | Silica | Synthetic |
| Cephascreen | Stago | Polyphenol | Rabbit brain cephalin |
| CK Prest | Stago | Kaolin | Rabbit brain cephalin |
| Pathromtin SL | Siemens | Silica | Vegetable |
| PTT-A | Stago | Silica | Rabbit brain cephalin |
| PTT-LA | Stago | Silica | Rabbit brain cephalin |
| SynthASil | HemosIL | Colloidal silica | Synthetic |
| Triniclot APTT-HS | Stago | Micronised silica | Purified (porcine and chicken) |
| Triniclot APTT-S | Stago | Micronised silica | Purified (porcine and chicken) |

**Table S2.** One-stage clotting assay results by aPTT reagent and analyser for samples spiked with rFIXFc at 0.80 IU/mL (Sample A), 0.20 IU/mL (Sample B) and 0.05 IU/mL (Sample C)

|  |  |  | **Sample A (nominal: 0.80 IU/mL)** | | | | | | **Sample B (nominal: 0.20 IU/mL)** | | | | | | **Sample C (nominal: 0.05 IU/mL)** | | | | | |
| --- | --- | --- | --- | --- | --- | --- | --- | --- | --- | --- | --- | --- | --- | --- | --- | --- | --- | --- | --- | --- |
| **APTT reagent** | **Coagulation analyser** | **n** | **Median (IU/mL)** | **Median recovery** | **Range** | **Mean (IU/mL)** | **Mean recovery** | **CV** | **Median (IU/mL)** | **Median recovery** | **Range** | **Mean (IU/mL)** | **Mean recovery** | **CV** | **Median (IU/mL)** | **Median recovery** | **Range** | **Mean (IU/mL)** | **Mean recovery** | **CV** |
| Actin FS | Siemens | 2 | 1.188 | 148.5% | 1.01–1.365 | 1.188 | 148.5% | 21.1% | 0.355 | 177.5% | 0.290–0.420 | 0.355 | 177.5% | 25.9% | 0.119 | 237.0% | 0.100–0.137 | 0.119 | 237.0% | 22.1% |
|  | Sysmex | 23 | 0.930 | 116.3% | 0.606–1.120 | 0.923 | 115.4% | 14.0% | 0.240 | 120.0% | 0.148–0.300 | 0.241 | 120.4% | 14.8% | 0.070 | 140.0% | 0.041–0.100 | 0.071 | 141.9% | 18.2% |
|  | Stago | 5 | 0.950 | 118.8% | 0.800–1.040 | 0.940 | 117.5% | 9.4% | 0.270 | 135.0% | 0.220–0.300 | 0.270 | 135.0% | 11.4% | 0.100 | 200.0% | 0.070–0.110 | 0.092 | 184.0% | 22.3% |
|  | All analysers | 30 | 0.945 | 118.1% | 0.606–1.365 | 0.944 | 118.0% | 15.1% | 0.244 | 122.0% | 0.148–0.420 | 0.253 | 126.7% | 18.8% | 0.070 | 140.0% | 0.041–0.137 | 0.078 | 155.3% | 25.6% |
| Actin FSL | Siemens | 7 | 0.912 | 114.0% | 0.548–1.010 | 0.862 | 107.7% | 18.3% | 0.310 | 155.0% | 1.155–0.350 | 0.276 | 137.8% | 26.5% | 0.078 | 156.0% | 0.030–0.120 | 0.077 | 154.6% | 43.0% |
|  | Sysmex | 4 | 0.929 | 116.1% | 0.730–1.120 | 0.927 | 115.9% | 18.2% | 0.275 | 137.5% | 0.200–0.304 | 0.264 | 131.8% | 18.6% | 0.075 | 150.0% | 0.058–0.103 | 0.078 | 155.5% | 24.6% |
|  | Stago | 1 | 0.860 | 107.5% | na | 0.860 | 107.5% | na | 0.250 | 125.0% | na | 0.250 | 125.0% | na | 0.090 | 180.0% | na | 0.090 | 180.0% | na |
|  | All analysers | 12 | 0.886 | 110.8% | 0.548–1.120 | 0.883 | 110.4% | 16.9% | 0.275 | 137.5% | 0.155–0.350 | 0.269 | 134.7% | 22.4% | 0.079 | 158.0% | 0.030–0.120 | 0.079 | 157.0% | 34.1% |
| Cephascreen | Stago | 6 | 0.774 | 96.8% | 0.670–0.973 | 0.800 | 100.0% | 12.9% | 0.223 | 111.5% | 0.220–0.268 | 0.232 | 116.2% | 8.2% | 0.062 | 123.0% | 0.060–0.080 | 0.065 | 130.7% | 12.2% |
| Pathromtin SL | Siemens | 5 | 0.890 | 111.3% | 0.847–0.940 | 0.894 | 111.7% | 4.7% | 0.320 | 160.0% | 0.310–0.363 | 0.330 | 164.9% | 7.1% | 0.096 | 192.0% | 0.030–0.120 | 0.083 | 166.0% | 49.9% |
|  | Sysmex | 4 | 0.685 | 85.6% | 0.640–0.730 | 0.685 | 85.6% | 5.4% | 0.185 | 92.5% | 0.177–0.210 | 0.189 | 94.7% | 7.9% | 0.063 | 125.0% | 0.050–0.070 | 0.061 | 122.5% | 16.8% |
|  | Stago | 2 | 0.715 | 89.4% | 0.710–0.720 | 0.715 | 89.4% | 1.0% | 0.235 | 117.5% | 0.220–0.250 | 0.235 | 117.5% | 9.0% | 0.058 | 115.0% | 0.050–0.065 | 0.058 | 115.0% | 18.5% |
|  | All analysers | 11 | 0.730 | 91.3% | 0.640–0.940 | 0.785 | 98.2% | 14.0% | 0.250 | 125.0% | 0.177–0.363 | 0.262 | 130.8% | 26.8% | 0.065 | 130.0% | 0.030–0.120 | 0.070 | 140.9% | 42.0% |
| SynthASil | IL | 31 | 0.810 | 101.3% | 0.420–0.926 | 0.798 | 99.8% | 11.5% | 0.230 | 115.0% | 0.110–0.300 | 0.226 | 112.8% | 16.3% | 0.068 | 136.0% | 0.020–0.100 | 0.068 | 135.3% | 23.5% |
|  | Sysmex | 6 | 0.645 | 80.6% | 0.570–0.860 | 0.668 | 83.5% | 16.0% | 0.160 | 80.0% | 0.140–0.200 | 0.165 | 82.5% | 15.7% | 0.040 | 80.0% | 0.030–0.060 | 0.040 | 80.0% | 27.4% |
|  | All analysers | 37 | 0.805 | 100.6% | 0.420–0.926 | 0.777 | 97.2% | 13.5% | 0.220 | 110.0% | 0.110–0.300 | 0.216 | 107.9% | 19.3% | 0.066 | 132.0% | 0.020–0.100 | 0.063 | 126.3% | 28.9% |
| APTT-SP | IL | 8 | 0.631 | 78.8% | 0.206–0.870 | 0.604 | 75.6% | 32.1% | 0.165 | 82.5% | 0.080–0.260 | 0.162 | 81.2% | 32.6% | 0.049 | 97.0% | 0.030–0.090 | 0.052 | 103.8% | 35.6% |
| Triniclot aPTT-S | Stago | 7 | 0.710 | 88.8% | 0.620–0.820 | 0.724 | 90.5% | 9.4% | 0.220 | 110.0% | 0.190–0.250 | 0.217 | 108.6% | 10.2% | 0.070 | 140.0% | 0.050–0.100 | 0.071 | 141.7% | 24.4% |
| Triniclot APTT-HS | IL | 2 | 0.838 | 104.7% | 0.740–0.935 | 0.838 | 104.7% | 16.5% | 0.252 | 126.0% | 0.210–0.294 | 0.252 | 126.0% | 23.6% | 0.074 | 147.0% | 0.060–0.087 | 0.074 | 147.0% | 26.0% |
|  | Sysmex | 1 | 0.650 | 81.3% | na | 0.650 | 81.3% | na | 0.200 | 100.0% | na | 0.200 | 100.0% | na | 0.060 | 120.0% | na | 0.060 | 120.0% | na |
|  | Stago | 5 | 0.620 | 77.5% | 0.599–0.760 | 0.661 | 82.6% | 10.9% | 0.190 | 95.0% | 0.177–0.237 | 0.202 | 100.9% | 13.6% | 0.070 | 140.0% | 0.055–0.087 | 0.073 | 146.8% | 17.8% |
|  | All analysers | 8 | 0.683 | 85.3% | 0.599–0.935 | 0.704 | 88.0% | 15.9% | 0.205 | 102.5% | 0.177–0.294 | 0.214 | 107.1% | 18.0% | 0.070 | 140.0% | 0.055–0.087 | 0.072 | 143.5% | 18.3% |
| PTT-A | Siemens | 2 | 0.544 | 67.9% | 0.477–0.610 | 0.544 | 67.9% | 17.3% | 0.167 | 83.3% | 0.143–0.190 | 0.167 | 83.3% | 20.0% | 0.042 | 83.0% | 0.040–0.043 | 0.042 | 83.0% | 5.1% |
|  | Stago | 20 | 0.613 | 76.6% | 0.380–0.720 | 0.594 | 74.3% | 15.2% | 0.170 | 85.0% | 0.097–0.220 | 0.167 | 83.7% | 21.1% | 0.042 | 83.0% | 0.020–0.090 | 0.047 | 94.7% | 39.9% |
|  | All analysers | 22 | 0.611 | 76.4% | 0.380–0.720 | 0.590 | 73.7% | 15.2% | 0.170 | 85.0% | 0.097–0.220 | 0.167 | 83.7% | 20.6% | 0.042 | 83.0% | 0.020–0.090 | 0.047 | 93.5% | 38.7% |
| PTT-LA | Siemens | 1 | 0.714 | 89.3% | na | 0.714 | 89.3% | na | 0.238 | 119.0% | na | 0.238 | 119.0% | na | 0.074 | 148.0% | na | 0.074 | 148.0% | na |
| CK Prest | Stago | 32 | 0.570 | 71.3% | 0.360–0.705 | 0.553 | 69.1% | 16.0% | 0.155 | 77.3% | 0.100–0.188 | 0.153 | 76.5% | 15.5% | 0.047 | 94.5% | 0.030–0.070 | 0.047 | 93.5% | 20.9% |
|  | IL | 1 | 0.445 | 55.6% | na | 0.445 | 55.6% | na | 0.125 | 62.5% | na | 0.125 | 62.5% | na | 0.037 | 74.0% | na | 0.037 | 74.0% | na |
|  | All analysers | 33 | 0.570 | 71.3% | 0.360–0.705 | 0.549 | 68.7% | 16.2% | 0.150 | 75.0% | 0.100–0.188 | 0.152 | 76.1% | 15.6% | 0.045 | 90.0% | 0.030–0.070 | 0.046 | 92.9% | 21.0% |
| **All OSC reagents** | **All analysers** | **175** | **0.720** | **90.0%** | **0.206–1.365** | **0.734** | **91.8%** | **24.5%** | **0.208** | **104.0%** | **0.080–0.420** | **0.209** | **104.4%** | **28.0%** | **0.060** | **120.0%** | **0.020–0.137** | **0.062** | **124.5%** | **35.0%** |

Siemens analysers included Models BCS and BCS-XP; Sysmex analysers included Models CA1500, CS2000, CS2500 and CS5100; Stago analysers included Models Evolution, STAR-Max and Compact; IL analysers included Models ACL TOP 500, 550, 700 and 750. aPTT: activated partial thromboplastin time; CV: coefficient of variation; IL: Instrument Laboratory; IU: international units; na: not applicable; OSC: one-stage clotting assay; rFIXFc: recombinant factor IX Fc fusion protein.

**Table S3.** Calibration frequency by analyser for samples contributed to the study

| **Analyser** | **Daily or with each run** | **Weekly** | **Every 3 weeks to 3 months** | **Every 6 months** | **Only with lot change or OOS controls** | **Not specified** |
| --- | --- | --- | --- | --- | --- | --- |
| Instrument Laboratory | 1 | 1 | 3 | 8 | 29 | 0 |
| Siemens | 0 | 0 | 0 | 3 | 12 | 1 |
| Sysmex | 7 | 8 | 3 | 2 | 17 | 1 |
| Diagnostica Stago | 22 | 0 | 10 | 3 | 42 | 1 |

OOS: out-of-specification.

**Table S4.** Number of dilutions performed by participating laboratories for samples contributed to the study

| **Sample** | **Single dilution** | **Two dilutions** | **Three dilutions** | **Not specified** |
| --- | --- | --- | --- | --- |
| Sample A (0.80 IU/mL) | 88 | 26 | 54 | 7 |
| Sample B (0.20 IU/mL) | 87 | 30 | 51 | 7 |
| Sample C (0.05 IU/mL) | 92 | 35 | 41 | 7 |

IU: international units.
